# Characterising urban green space density and footpath-accessibility in models of BMI

**DOI:** 10.1186/s12889-020-08853-9

**Published:** 2020-05-24

**Authors:** Philip Carthy, Sean Lyons, Anne Nolan

**Affiliations:** 1 Economic and Social Research Institute, Whitaker Square, Sir John Rogerson’s Quay, Dublin 2, Ireland; 2 Department of Economics, Trinity College Dublin, Dublin, Ireland; 3 The Irish Longitudinal Study on Ageing, Trinity College Dublin, Dublin, Ireland

**Keywords:** Body mass index, Urban green space, Footpaths, Older adults, Ireland

## Abstract

**Background:**

While exposure to urban green spaces has been associated with various physical health benefits, the evidence linking these spaces to lower BMI, particularly among older people, is mixed. We ask whether footpath availability, generally unobserved in the existing literature, may mediate exposure to urban green space and help explain this volatility in results. The aim of this study is to add to the literature on the association between urban green space and BMI by considering alternative measures of urban green space that incorporate measures of footpath availability.

**Methods:**

We conduct a cross-sectional study combining data from The Irish Longitudinal Study on Ageing and detailed land use information. We proxy respondents’ exposure to urban green spaces at their residential addresses using street-side and area buffers that take account of the presence of footpaths. Generalised linear models are used to test the association between exposure to several measures of urban green space and BMI.

**Results:**

Relative to the third quintile, exposure to the lowest quintile of urban green space, as measured within a 1600 m footpath-accessible network buffer, is associated with slightly higher BMI (marginal effect: 0.80; 95% CI: 0.16–1.44). The results, however, are not robust to small changes in how green space is measured and no statistically significant association between urban green spaces and BMI is found under other variants of our regression model.

**Conclusion:**

The relationship between urban green spaces and BMI among older adults is highly sensitive to the characterisation of local green space. Our results suggest that there are some unobserved factors other than footpath availability that mediate the relationship between urban green spaces and weight status.

## Background

Obesity has become a major international public health challenge. Globally, its prevalence, as measured by a body mass index (BMI) ≥ 30 kg/m^2^, is estimated to have risen from 3.2 to 10.8% in men and from 6.4 to 14.9% among women between the years 1975 and 2014 [1]. In Ireland, in 2015, 23% of the adult population were classified as obese [2].^1^ High BMI is a known risk factor for various non-communicable diseases, including cardiovascular disease, [4] diabetes, [5] heart disease and stroke [6]. This upward trend could thus create a significant burden on healthcare systems across the world. While its cause is undoubtedly multifaceted [7], it is possible that the form of the modern built environment has a role in promoting negative health behaviours that ultimately result in adiposity [8]. Several aspects of the urban environment might be relevant, including land use mix, the extent of urban sprawl, the food environment, crime, walkability, and access to green spaces [9]. Given that two-thirds of the world’s population is expected to live in urban areas by 2050 [10], it is important that research aims to understand the interconnections between urban living and health behaviours. Of particular interest in the current work is the potential association between weight status and the availability of pedestrian-accessible urban green spaces.

While many studies have identified positive associations between urban green space and various dimensions of individual health [11, 12], the evidence linking greenness to decreased obesity rates remains equivocal. A recent review of the literature by Browning & Lee [13] find that just 50% of reviewed analyses (*n* = 26) produce significant results in favour of a green space-obesity link. Indeed, some counterintuitive positive associations have also been found [14]. The literature which specifically looks at associations between urban green space and obesity among older people remains limited but is equally divided. Using a large sample of those aged 45 and over in Australia, Astell-Burt et al. [15] find that higher exposure to urban green space is associated with reduced risk of obesity among women but that the protective effect is absent for men. Li et al. [16] find no association between green spaces and adiposity in a US-based sample of people aged 50–75. Using Irish data, Dempsey et al. [17] find a u-shaped relationship between urban green space and obesity in older adults, with those receiving the lowest and highest exposures to green space in the vicinity of their residential address exhibiting an increased probability of being obese.

The apparent conflict in the existing evidence could be attributable to various methodological concerns: over-reliance on cross-sectional data [18], absence of objective obesity measurements in some studies, use of aggregate rather than individual-level data, or insufficient control for potentially confounding factors [11, 12]. We posit that a further, relatively unexplored issue might also be relevant. That is, while standard approaches used to objectively measure urban greenness generally quantify the *availability* of green spaces, they often disregard the issue of *accessibility* of the same spaces to individual study participants. Previous evidence suggests that the primary channel through which green spaces may affect health is by facilitating physical activity [11, 12]. Therefore, it is likely that spaces need to be easily accessible to the target population in order to effectively promote positive health behaviours. As such, the interaction between green spaces and local footpath networks may be of particular relevance. For example, living in a locality with extensive green coverage may not be associated with any physical health benefits if the same area lacks footpaths to access the green spaces on foot. Conversely, an area which has sufficient footpath access to a limited set of green spaces may effectively promote physical activity and accrue health benefits for residents, despite the fact that it is observationally ‘less green’.

The paper builds on an earlier paper by Dempsey et al. [17] that found that, among the over 50s in Ireland, those with the lowest and highest exposures to green space in the vicinity of their residential address had an increased probability of obesity. One potential explanation for the counterintuitive results at the higher quintiles of green space exposure is that the study did not consider the availability of footpaths. The aim of this study is, therefore, to further investigate the association between urban green space and BMI by explicitly controlling for footpath availability in urban areas, in the same setting explored by Dempsey et al. We exploit a novel data source that combines individual-level geocoded survey microdata with detailed land-use information from which the density of both local urban green spaces and footpaths can be extracted. While the analysis does rely on cross-sectional methods, the data source contains objective BMI measurements as well as a wealth of information on variables which may confound the relationship between urban green spaces and obesity. Our analysis thus overcomes many of the methodological challenges cited above.

## Methods

This paper combines two distinct datasets in order to examine the relationship between urban green space and BMI: The Irish Longitudinal Study of Ageing, and a land-use database known as Prime2. The datasets and the methods used to link and analyse them are outlined below.

### The Irish longitudinal study on ageing (TILDA)

TILDA is a nationally representative survey of those aged over 50 in the Republic of Ireland. Data for Wave 1 (W1), which forms the basis of the analysis in the current study, was initially collected between October 2009 and July 2011. During this period, 8175 individuals from a sample of 6279 households were recruited to participate in the study. Respondents’ spouses and partners were also invited to participate, regardless of their age, and so the full W1 sample size is 8504. The data were primarily collected using Computer Assisted Personal Interviewing (CAPI) carried out by trained interviewers, face-to-face at each individual’s home. Sensitive questions were included in a supplemental self-completed questionnaire (SCQ), which respondents returned by mail. Wave 1 respondents were also invited to attend a nurse-administered health assessment at a dedicated centre or, where attendance was infeasible or impractical, to complete a modified partial assessment in the home. Follow-up data have been collected at two-year intervals [19, 20] but are not used here.

TILDA recruitment followed the RANSAM protocol [21], a method which samples households from the population of residential addresses in the Republic of Ireland. The geo-location of each respondent’s residential address is thus known and can form the basis of spatial links to additional external data sources.

#### Outcome: body mass index (BMI)

**Fig. 1:**
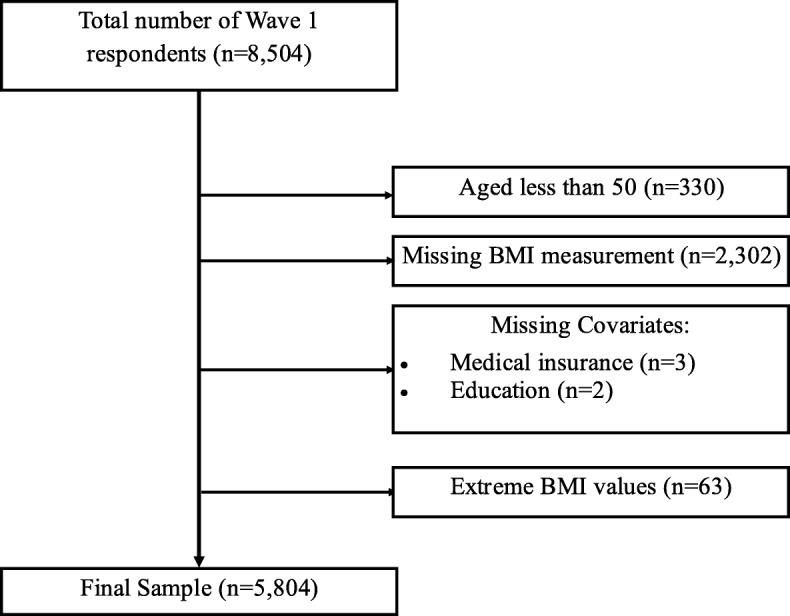
Construction of the final sample

BMI, calculated as a person’s weight in kilograms divided by the square of their height in metres (*kg*/*m*^2^), serves as the health outcome of interest in this paper. The index is widely used as a tool to classify adult obesity based on the cut-off values defined by the World Health Organization [22]. Self-reported measures of height and weight are subject to measurement error [11, 12], and so we use objective measurements of height and weight that were collected as part of the TILDA health assessment. After each participant had removed footwear and any heavy outer garments, SECA 240 wall mounted rods, and SECA electronic floor scales were used to record height and weight, respectively [23]. Since the health assessment was an optional component of the study, a valid BMI measurement is unavailable for 2302 respondents in our sample, necessitating their exclusion.^2^ The those with a BMI more than three standard deviations from the mean of the distribution (*n* = 63) are excluded from the analysis as the recorded values appear biologically implausible. See Fig. 1 for full details on how the final sample was constructed. The distribution of BMI values among TILDA respondents in this final sample is presented in Fig. S1 in the Additional file 1. The observed range of BMI scores is 15.88–43.89, with a mean value of 28.45.

#### Additional control variables

The geography of urban green spaces may be systematically associated with socioeconomic characteristics [24]. In particular, those with favourable economic circumstances may have the ability to self-select into more attractive and potentially greener neighbourhoods [25]. While the structure of our combined data source does not allow us to capture all such factors, the richness of the TILDA dataset allows us to control for many socioeconomic, demographic, and health-related factors that may jointly determine BMI and exposure to green space. Importantly, we control for income category in all our econometric models. Failure to do so could lead to overestimation of a positive relationship between greenness and health [13]. Our full set of control variables closely follows Dempsey et al. [17] and includes age category, urban location, gender, income category, employment status, marital status, highest level of educational attainment, medical cover, smoking status, and a dummy variable that indicates reported difficulty walking 100 m. Descriptive statistics for these variables appear in Table 1.

**Table 1:** Descriptive statistics

		Frequency	Percent
**Green Space** *(1600 m Network)*	Non-urban Settlement	3561	61.35
	Quintile 1A (lowest)	449	7.74
	Quintile 2A	449	7.74
	Quintile 3A	450	7.75
	Quintile 4A	447	7.70
	Quintile 5A (highest)	448	7.72
**Urban Location**	Non-Dublin	4288	73.88
	Dublin	1516	26.12
**Gender**	Male	2672	46.04
	Female	3132	53.96
**Age Category**	50–64	3462	59.65
	65–74	1548	26.67
	≥ 75	794	13.68
**Income Category**	0–9999	426	7.304
	10,000 - 19,999	1009	17.38
	20,000 - 39,999	1944	33.49
	40,000 - 69,999	1236	21.3
	≥ 70,000	560	9.65
	Not reported	629	10.84
**Marital Status**	Married	4197	72.31
	Never married	471	8.12
	Sep/divorced	387	6.67
	Widowed	749	12.9
**Employment Status**	Employed	2209	38.06
	Retired	2144	36.94
	Other	1451	25.00
**Smoker**	Never	2606	44.9
	Past	2266	39.04
	Current	932	16.06
**Educational Attainment**	Primary/none	1519	26.17
	Secondary	2371	40.85
	Third/higher	1914	32.98
**Medical Cover**	Not covered	588	10.13
	Medical insurance	2631	45.33
	Medical card	2585	44.54
**Mobility**	No difficulty walking 100 m	5456	94.00
	Difficulty walking 100 m	348	6.00
Total		5804	100.00

Consistent with the overall cohort, females are slightly over-represented, making up 54% of our final sample [23]. Despite TILDA’s focus on older people, the W1 cohort is relatively young and active in the labour market, with 59.7% of the sample under the age of 65 and 38% in employment at the time of interview. A broad spectrum of educational attainment and income levels are captured in the data. Smoking habits are prevalent among the cohort with past and current smokers combined accounting for 55.1% of respondents. Mobility-limiting disabilities are relatively uncommon at W1, with 6.1% indicating that their ability to walk 100 m would be impeded by some physical or mental health condition. Nevertheless, it is important to control for such difficulties as the relationship between greenness and BMI is likely mediated by an ability to access and utilise the relevant spaces.

### Land use data: Prime2

**Fig. 2:**
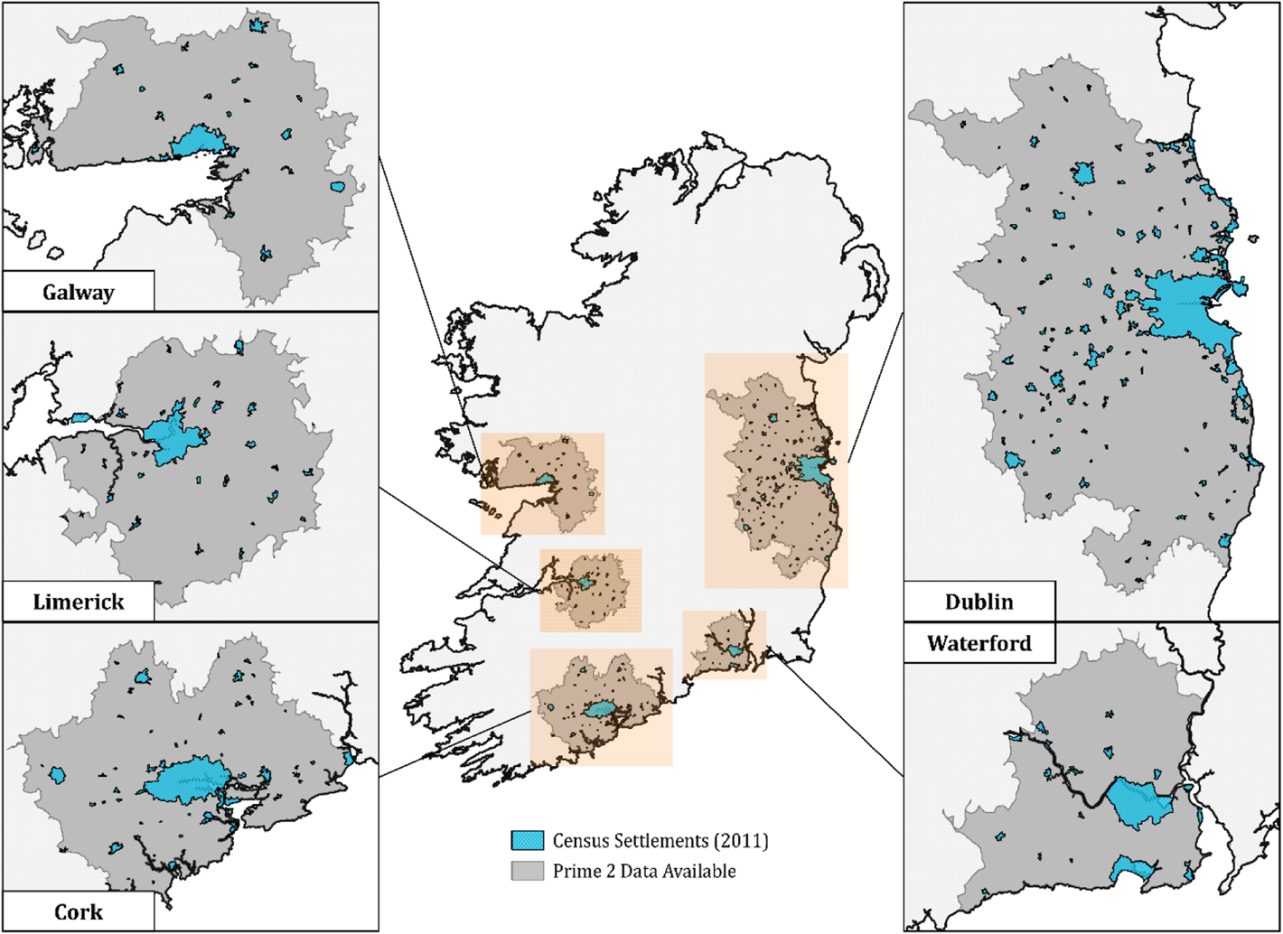
Map of Ireland indicating regions in which `urban` green space is analysed in this paper. Source: analysis by the authors prepared using QGIS 3.4 (under the GNU General Public License) with Prime2 data described in the text; and the map of settlements is from Central Statistics Office Census 2011 Boundary Files (https://www.cso.ie/en/census/census2011boundaryfiles/)

The spatial information used to derive the amount of urban green space in the vicinity of TILDA residential addresses is drawn from ‘Prime2’, an object-oriented digital mapping model which standardises a wealth of spatial data for Ireland. The dataset was developed by Ordnance Survey Ireland (OSI), the country’s national mapping agency. Prime2 includes three features that are particularly relevant to the current study: 1) a detailed land-use data from which green areas can be identified, 2) a fully connected road network from which the theoretical accessibility of green areas can be imputed, and 3) a complete (albeit disjoint) set of urban footpaths from which the feasibility of walking along a particular route may be approximated. Walkable footpaths are taken to include the set of paths labelled as Sidewalks, Boardwalk, Walk general, Pedestrian Zone, Walk unmarked and Towpath. They exclude those defined as Pedestrian bridge, Pedestrian plaza or Steps, not all of which are accessible to pedestrians. Footpaths within parks are not available in the dataset. Data covering extensive areas surrounding the country’s five primary urban centres (Dublin, Cork, Galway, Limerick, Waterford) were made available for the purposes of the current study. These areas, however, contain large commuting zones that may be quite rural in nature. We calculate various dimensions of green space footpath-accessibility in regions identified as ‘urban settlements’ in the 2011 Irish Census.^3^ Figure 2 provides a map of the areas considered ‘urban’ in the analysis.

#### Characterising local green space

The strategy we employ to determine greenness of each urban TILDA respondent’s locality builds on existing methods from the literature with the specific aim of accounting for urban accessibility factors, which may be omitted under traditional research designs. Broadly, we use Geographic Information Systems (GIS) to define a buffer zone around each respondent’s residential address, and subsequently calculate the share of land area within the buffer that is made up of green spaces as a measure of exposure.^4^ It is ultimately an empirical question how best to specify these buffer zones such that the green space metric captures what has the greatest potential relevance to respondents’ health outcomes. Indeed, past research has shown that observed associations between greenness and health can be sensitive to researchers’ choice of green space characterisation [8].

**Fig. 3:**
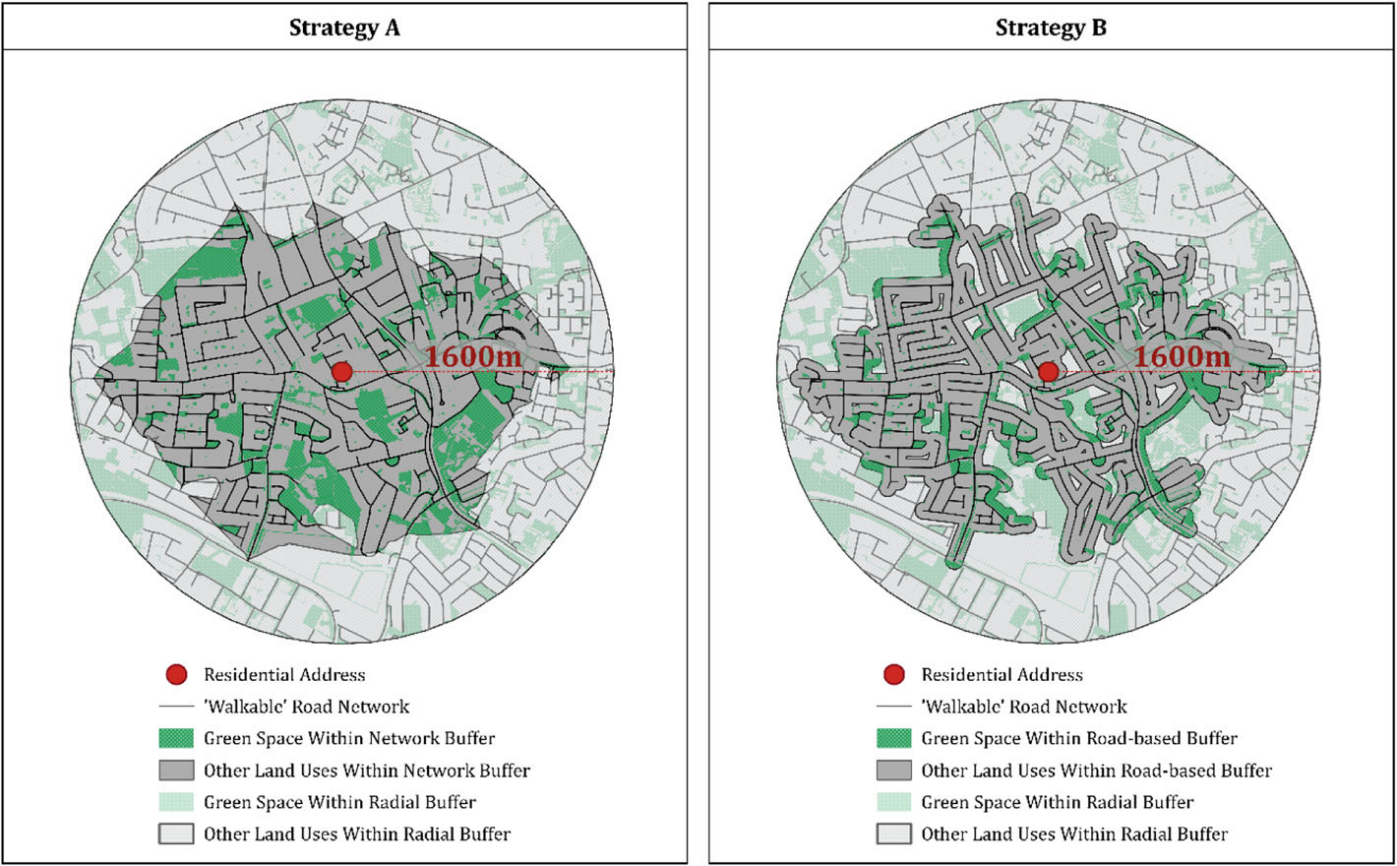
Comparison of network and street-side buffer strategies. Source: analysis by the authors prepared using QGIS 3.4 with Prime2 data described in the text

Basing the analysis on circular buffers ignores various dimensions of connectivity within the urban space and may misrepresent the extent of the area that can be reached by a respondent on foot. For example, if the urban landscape does not offer a straight-line path between the buffer centre and its edge, then an individual wishing to travel between the two locations necessarily transverses a distance greater than the buffer radius. In such cases, a circular buffer can capture green space that lies beyond an assumed maximum walking distance from the residential address. This issue is accentuated in regions where urban layouts do not follow grid systems (as is the case in Ireland) since straight-line paths between locations are generally uncommon. To overcome this issue, we follow a number of recent studies, which have carried out green space analysis within network buffers [27–30]. Such buffers are drawn based on a maximum distance travelled across a road network (See Panel A of Fig. 3).

While network buffers offer an improved characterisation of the maximum pedestrian-accessible area around a given residential address, they cannot account for all accessibility issues within the chosen buffer space. For example, it may be impractical to walk along certain roads even when they are proximal to one’s residential address. To this issue, we offer a novel solution. We produce network buffers using only roads with which footpaths are associated. Specifically, a junction-to-junction road segment is only included in a network buffer in this study if a set of footpaths, with a combined length which exceeds half that of the road segment, can be identified within 25 m of the road segment centreline. As a result, our analysis is restricted to geographic areas where the density of local footpaths is high and, on average, green spaces that are not accessible on foot are excluded. A more formal description of our methodology is provided in the Additional file 1.

Even within these areas, which we term ‘footpath-accessible network buffers’, the proximity of green space to the road network itself might have a mediating role in any association between greenness and health. For example, recent work has identified explicit associations between street-side greenery and health outcomes [35]. To test the relevance of such greenery (e.g., green common areas in housing estates) in our context, we define second set of buffer zones which restrict the classification of relevant green spaces to those that fall within 50 m of roads provided with footpaths (See Panel B of Fig. 3). A comparison of results using the two alternative buffer definitions will allow us to identify which set of green spaces, if any, is most associated with BMI.

The appropriate size to draw the buffers is also unclear. A recent survey of the literature by Browning & Lee [13] suggests that, on average, larger buffers sizes (up to 2000 m) best predict dimensions of physical health, but that for studies which centre the zones on exact residential addresses (as is the case in the current study), this predictive power might plateau at a much smaller buffer size (500-1000 m). Since our observed results may be sensitive to this choice, we perform our analysis using multiple buffer extents. Our main specification follows Dempsey et al. [17] in using a 1600 m buffer, which creates a zone roughly appropriate for a 20-min walk from one’s home address. We then repeat the statistical analysis with a smaller 800 m buffer.

Our final analysis thus utilises four varied characterisations of local green space: “Footpath-accessible network buffers” covering 1600 m and 800 m spaces and “footpath-accessible street-side buffers” of the same sizes. In order to preserve the anonymity of individual TILDA respondents, the final variables enter our statistical models in categorical form. Specifically, the variables used represent the quintile of green space exposure which a respondent receives. The correlations among these measures are shown in Table 2 for the 1600 m metrics. The correlation between street-side and network buffers is high; that between these metrics and circular buffers is lower. Respondents who reside in non-urban settlement areas are coded as a separate category to allow a larger sample to be used, permitting more precise estimation of non-green space control variables.

**Table 2:** Spearman rank correlations for green space quintiles, comparing 1600 m circular, network and street-side buffers

	Circular buffer	Network buffer	Street-side buffer
Circular buffer	1.00		
Network buffer	0.697	1.00	
Street-side buffer	0.641	0.841	1.00

### Model

We test the association between urban green space and BMI using regression techniques, specifically, using a generalised linear model (GLM). The GLM framework offers additional model flexibility compared to traditional Ordinary Least Squares (OLS) and is employed when the distribution of the outcome variable may not be normal. In particular, the researcher may specify a functional form that links the outcome variable to a linear index of explanatory variables and make a distributional assumption about the variance of the estimator. The model, as it applies to the current context, is as follows:

1 $$ g\left({BMI}_i\right)={\beta}_0+{\beta}_1{green}_i+\sum {\beta}_k{\boldsymbol{X}}_{ki} $$

2 $$ Var\left[{BMI}_i{\left|{green}_i,\boldsymbol{X}\right.}_{ki}\right]\propto {\left(\boldsymbol{E}\left[{BMI}_i\left|{green}_i,{\boldsymbol{X}}_{ki}\right.\right]\right)}^v $$

where *g*(.) is a function that links BMI to our independent variables of interest, *green*_*i*_ is a categorical representation of local green space for individual *i*, and the ***X*** represents the vector of *k* socioeconomic and health-related control variables discussed above. We perform a specification search to identify the most appropriate functional form for *g*(.) (link function) and value for *v* (estimator family). In the search process, we allow the link to be the identity (linear), natural log, and square root functions, and *v* = 0, 1, 2 (equivalent to Gaussian, Poisson, and Gamma families respectively). The variance of the dependent variable (*Var*) is assumed to vary (∝) according to some function of the mean of the variable. The models chosen are those with the lowest Akaike’s Information Criterion (AIC) [32] and Schwarz Bayesian Criterion (BIC) [33]. The Gaussian family model with identity link function emerges as the most efficient. As such, the results reported are equivalent to those produced by a linear OLS model. Robust standard errors, clustered at the household level, are computed to allow for a general form of heteroscedasticity. We run two specifications of each model; one with the full set of covariates as set out above, and a second “parsimonious” model with groups of covariates that are collectively insignificant at the 5% level excluded. This allows us to check the sensitivity of the urban green space coefficients to the set of covariates chosen for inclusion.

## Results

**Fig. 4:**
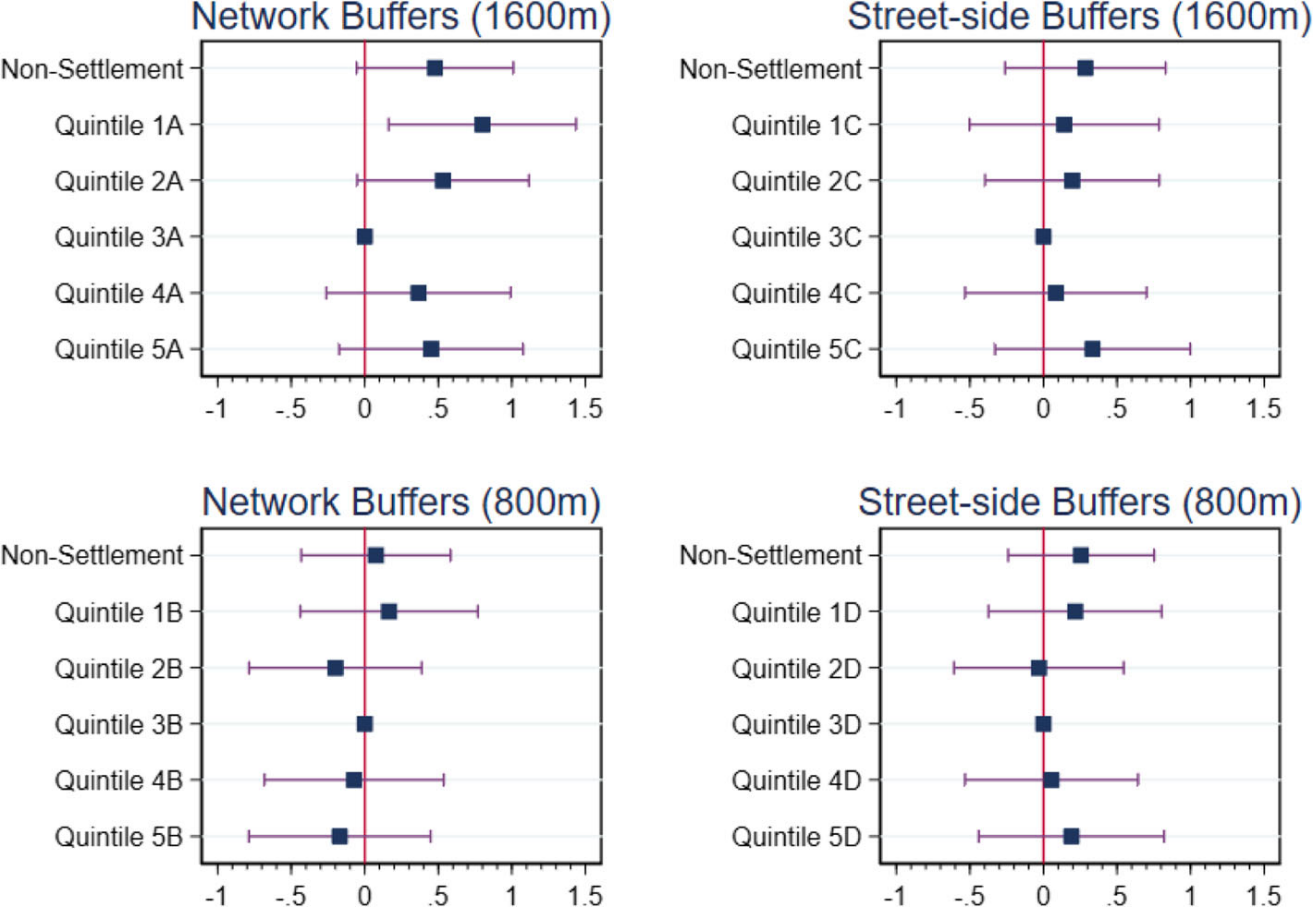
GLM regression results. Marginal effects of footpath-accessible green space quintile (relative to 3rd quintile) on BMI, comparing street-side and network buffers at 800 m and 1600 m. Notes: The values along the x-axis refer to marginal effects. Horizontal bars represent 95% confidence intervals. Quantile 1 refers to the lowest quintile of footpath-accessible green space, while quintile 5 refers to the highest. A-D represent the measure of green space footpath-accessibility used (see also Table 2)

Table 3 presents the results of the estimated GLMs and Fig. 4 displays the marginal effects and 95% confidence intervals for the estimates.^5^ Model 1, which characterises local green space using 1600 m footpath-accessible network buffers, shows a u-shaped relationship with BMI, although the estimates for higher quintiles are statistically insignificant. Higher BMI scores are observed among those living in areas with low exposure to footpath-accessible green space. Relative to living in the third quintile, exposure to the first (lowest) quintile of footpath-accessible green space under this definition is associated with an increase in BMI of 0.80. As the mean BMI value in this sample is 28.45, this estimate equates to a 2.8% increase in BMI. By way of comparison, the effect of having a primary-level education (relative to a second-level education) is 0.49 or 1.7%. Although we observe a positive relationship between quintiles 4 and 5 (highest) and BMI, these coefficients are not statistically significant. The inclusion of footpath-accessibility measures into a 1600 m network buffer thus weakens the positive association between high exposure to green space and BMI found by Dempsey et al. [17] but does not completely remove the pattern. Indeed, it is noteworthy a more parsimonious version of this model (Model 2) which drops groups of covariates that are collectively insignificant at the 5% level, produces a slightly stronger u-shaped relationship with marginal statistical significance observed on the quintile 4 and 5 coefficients. As shown in Additional file 1: Table S2, Model 2 drops Dublin location, income, marital status and medical cover from the covariates included in Model 1.

**Table 3:** Results using footpath-accessible network and street-side buffers

		Network Buffer				Street-side Buffer			
		(1)	(2)	3)	(4)	(5)	(6)	(7)	(8)
		Marginal Effect (SE)	Marginal Effect (SE)	Marginal Effect (SE)	Marginal Effect (SE)	Marginal Effect (SE)	Marginal Effect (SE)	Marginal Effect (SE)	Marginal Effect (SE)
**Share of Footpath-accessible Green Space**									
**1600 m Network Buffer**	Non-Urban Settlement	0.478* (0.272)	0.722*** (0.238)						
	Quintile 1A (lowest)	0.800** (0.325)	0.722** (0.323)						
	Quintile 2A	0.533* (0.298)	0.486 (0.299)						
	Quintile 3A	[ref.]	[ref.]						
	Quintile 4A	0.366 (0.320)	0.435 (0.320)						
	Quintile 5A (highest)	0.452 (0.319)	0.532* (0.318)						
**800 m Network Buffer**	Non-Urban Settlement			0.0764 (0.259)	0.269 (0.226)				
	Quintile 1B (lowest)			0.166 (0.308)	0.128 (0.306)				
	Quintile 2B			−0.199 (0.300)	−0.226 (0.298)				
	Quintile 3B			[ref.]	[ref.]				
	Quintile 4B			−0.0724 (0.311)	−0.0200 (0.312)				
	Quintile 5B (highest)			−0.170 (0.315)	−0.0739 (0.313)				
1600 m Street-side Buffer	Non-Urban Settlement					0.285 (0.278)	0.466* (0.240)		
	Quintile 1C (lowest)					0.141 (0.329)	0.0909 (0.322)		
	Quintile 2C					0.195 (0.302)	0.201 (0.301)		
	Quintile 3C					[ref.]	[ref.]		
	Quintile 4C					0.0848 (0.315)	0.148 (0.312)		
	Quintile 5C (highest)					0.334 (0.338)	0.449 (0.331)		
800 m Street-side Buffer	Non-Urban Settlement							0.256 (0.253)	0.427** (0.214)
	Quintile 1D (lowest)							0.215 (0.300)	0.193 (0.297)
	Quintile 2D							−0.0314 (0.294)	−0.0408 (0.294)
	Quintile 3D							[ref.]	[ref.]
	Quintile 4D							0.0550 (0.300)	0.128 (0.300)
	Quintile 5D (highest)							0.190 (0.321)	0.309 (0.311)
**N**		5804	5807	5804	5807	5804	5807	5804	5807

Standard errors in parentheses. * *p* < 0.1 ** *p* < 0.05 *** *p* < 0.01.

The results in column (1) refer to the results of the full model with green space footpath-accessibility expressed in terms of a 1600 m network. The results in column (2) are those of the parsimonious specification. The parsimonious models have three more observations due to the medical insurance variable being dropped. The results in Column (3) are from full model with green space footpath-accessibility expressed in terms of an 800 m network buffer, while the results in Column (4) refer to the more parsimonious specification of the models. Columns (5) to (8) are the equivalent models using green space footpath-accessibility using street-side buffers.

As noted earlier, these models place non-urban residents in a separate green space category. The pattern of marginal effects on the urban green space variables is broadly similar when these observations are excluded from the analysis (see Additional file 1: Table S4). In addition, we considered an alternative formulation of the dependent variable based on the probability of having a BMI > =30 (obesity) using a limited dependent variable model. None of the green space categories has a statistically significant marginal effect in this model. See Additional file 1: Table S5.

Our results differ from the existing evidence more significantly when using other characterisations of footpath-accessible local green space. Model 3, which measures green space in an 800 m network buffer, suggests no significant differences in BMI values across quintiles of green space exposure. Similarly, while the observed coefficients from models which measure green space with footpath-accessible street-side buffers (Models 5–8) broadly follow a u-shape, the differences in BMI scores across quintiles of street-side greenness are not statistically significant.

## Discussion

Overall, we find no clear association between various measures of footpath-accessible urban green space and BMI. In addition, our results emphasise the sensitivity of existing results in the literature to the characterisation of green space. While we do find that estimated exposure to the lowest quintile of green space in a 1600 m footpath-accessible network buffer is associated with higher BMI scores, it is clear that an adjustment for footpath-accessibility of urban green space, as we have defined it, has not offered a complete explanation for the u-shaped relationship previously identified in these data by Dempsey et al. [17].

In this context, it remains possible that other unobserved elements of the urban environment, or indeed of green spaces themselves, may affect the individual decision to utilise green areas for physical activity. For example, inadequate lighting, restricted opening hours, or the presence of anti-social behaviour may, at times, impede usage of some spaces. Equally, the decision to use green spaces may be driven by individual preferences that cannot be captured through analysis of the urban environment alone [34]. It remains for future work to incorporate such hypotheses into an analysis of accessible green space. In addition, there may be explanations for any observed link between green space and BMI that operate via mechanisms other than the promotion of physical activity such as stress reduction, increased social interaction, etc. [12].

It is also striking that adjustments to the extent of the area in which green space is measured can substantially alter, and in our context statistically nullify, the association with BMI. Given that our green-space variables are correlated with each other (see Table 2), it is perhaps unsurprising that a u-shaped relationship broadly remains across most of our specifications. It is, however, interesting that statistical significance depends on the exact characterisation used. While such volatility in results is not unusual within the literature [11, 13], it serves to reaffirm the sensitivity of findings in this area to research design choices. Previous research on the association between green space and obesity risk and physical activity in the Netherlands, albeit using different measurements of green space, also found that the association was sensitive to the types of measurements used [31]. Our results are also consistent with a recent review of the literature [13] which suggests the size of the area in which green space is measured can meaningfully alter the strength of its associations with health outcomes. However, to our knowledge, this is the first study to incorporate measures of footpath availability in a study of the association between local urban green space and BMI. The question of how best to characterise local green space such that the definitions are those with the greatest possible relevance to individual behaviour and ultimately health outcomes remains broadly unanswered and should also be further addressed in future work.

The current study is subject to several limitations, primarily related to the green space exposure metrics used in the analysis. First, our dataset omits footpaths within parks, which probably implies that our measures of footpath-accessible green space underestimate the green space exposures of respondents living in close proximity to parks. This problem is mitigated by the categorical representation of green space in the models: most respondents living beside a park will be in a high exposure quintile anyway. Nevertheless, it is possible that some respondents were placed in a lower exposure category due to this omission. Second, our key independent variables capture elements of both availability and accessibility. Longitudinal data incorporating changes to accessibility over time (e.g., new footpaths, greater opening hours, etc.) offers one approach that could be considered in future work. Second, the process of building a ‘walkable’ road network based on proximity to footpaths is one which undoubtedly contains at least some measurement error. It is possible that some road segments excluded because of a lack of identifiable footpath may actually be walkable. This, in turn, could exclude some green spaces from our analysis. Conversely, our data lack detailed descriptions of individual footpaths, so our analysis can say little about the quality of the footpath network used. It is plausible that some areas treated as footpath-accessible in our data could contain poor-quality paths on which it would be impractical for an older person to walk. This, in turn, may lead to an overestimate of green space accessibility in the affected areas. More generally, we cannot rule out the possibility that there is an effect on BMI from walking on these footpaths that is separate to that operating via access to green spaces. In addition, the measures of footpath-accessibility developed in this paper utilise green spaces that are proximal to the public road network. Given the current data, we do not observe the ownership of these green spaces. Some green areas that lie within a respondent’s footpath-accessible buffer zone may not be available for public use. This could also lead to an overestimation of green space exposure for some respondents in our analysis. Finally, as our green space and walkability measurements are drawn from a specific database developed and maintained in Ireland, it is hard to directly compare our empirical results with those from other studies. If sufficiently high-resolution data on green space and footpath networks were available with international coverage using consistent metrics (e.g. the normalized difference vegetation index (NDVI) for green space characterisation), that should allow better ease of comparison across national samples.

Two broader limitations are also noteworthy: first, it is unclear whether any measure of green space based on residential addresses can be considered an accurate proxy for the exposure the resident receives. Exposure to green space may instead be determined by unobserved dimensions of one’s lifestyle. For example, if a respondent as a particular preference for spending time in green spaces, they may be willing to use other forms of transport to travel to spaces that are beyond walking distance from their home. Equally, if a respondent’s routine includes activities that take place away from their reported residential address, then the area in which we measure green space may not be the most relevant. Second, since we only observe land use data at one point in time, we are precluded from using the longitudinal dimension of TILDA in our analysis. We cannot, therefore, fully account for the possibility that respondents systematically self-select into areas with specific levels of green space exposure. No causality can be assigned to the results presented in this paper. Finally, a key potential mechanism linking green space exposure and BMI is physical activity [11, 12]; the absence of objectively-measured physical activity data for this sample of TILDA respondents means that we cannot investigate the impact of different conceptualisations of green space accessibility on physical activity.

## Conclusions

The relationship between urban green spaces and BMI among older adults is highly sensitive to the characterisation of local green space. This study contributes to the literature on the association between green space and BMI by considering alternative definitions of urban green space that incorporate footpath availability. Our results suggest that there are some unobserved factors other than footpath availability that mediate the relationship between urban green spaces and weight status.

We find suggestive evidence that being exposed to lower levels of green space, as proxied by a 1600 m footpath-accessible network buffer centred on one’s residential address, is associated with increased BMI. However, the association loses statistical significance if the buffer size is reduced to 800 m or if green areas that are located adjacent to walkable roads are used, despite relatively high correlations among respondent exposure rankings using the various buffer types. While the associations we report are not statistically significant in most cases, our model coefficients do broadly follow a u-shape, consistent with previous work carried out by Dempsey et al. [17] in a similar context. This suggests that the incorporation of footpath availability measures into the analysis does not offer a full explanation for their results. We suggest that future work could include additional features of the built environment or dimensions of individual preferences for green space usage in a similar analysis.

## Supplementary information

**Additional file 1.**

## Data Availability

Data may be obtained from a third party and are not publicly available. The linked data file can be accessed on-site via the TILDA hot desk system (contact tilda@tcd.ie for details). The unlinked data file can be accessed from the Irish Social Science Data Archive (www.ucd.ie/issda) and other sources, e.g. the Gateway to Global Aging (www.g2aging.org) and the Interuniversity Consortium for Political and Social Research (www.icpsr.umich.edu/icpsrweb/).

## Abbreviations

TILDA: The Irish Longitudinal Study on Ageing
BMI: Body Mass Index
W1: Wave 1
GLM: Generalised Linear Model
OSI: Ordnance Survey Ireland

## Footnotes

1 Comparable data from the Healthy Ireland Survey for 2015 indicate that approximately one third of the older (55+) population were obese [2]. A comparison of rates of obesity among the over 50s in Ireland, England and the US showed that rates of obesity were similar in Ireland and England, and considerably lower in both countries than in the US [﻿3﻿].

2 Our sample represents 73% of TILDA wave 1 respondents, Those with more education, in better health and in the youngest age groups were more likely to complete the TILDA health assessment [23].

3 Specifically, the green space surrounding a TILDA residential address is characterised if a) the address is located within a cluster of at least 50 occupied dwellings; b) the cluster contains is clear evidence of an urban centre; c) the distance to the next nearest occupied dwelling does not exceed 100 m [26].

4 Green spaces are derived from the vegetation layer of PRIME2. The various land uses that this layer incorporates are detailed in Table A1 in Additional file 1.

5 Results for the full set of covariates are presented in Tables A2 and A3 in Additional file 1.
