# Supplementary material for: Characterising urban green space density and footpath-accessibility in models of BMI
Source: BMC Public Health. 2020 May 24;20:760. doi: 10.1186/s12889-020-08853-9 (PMC7245785; doi:10.1186/s12889-020-08853-9)
Supplement: Supplementary file 1 — Additional file 1. [file 12889_2020_8853_MOESM1_ESM.docx]

# Additional file 1

**Figure S1: Frequency distribution of BMI values among the TILDA sample**

**
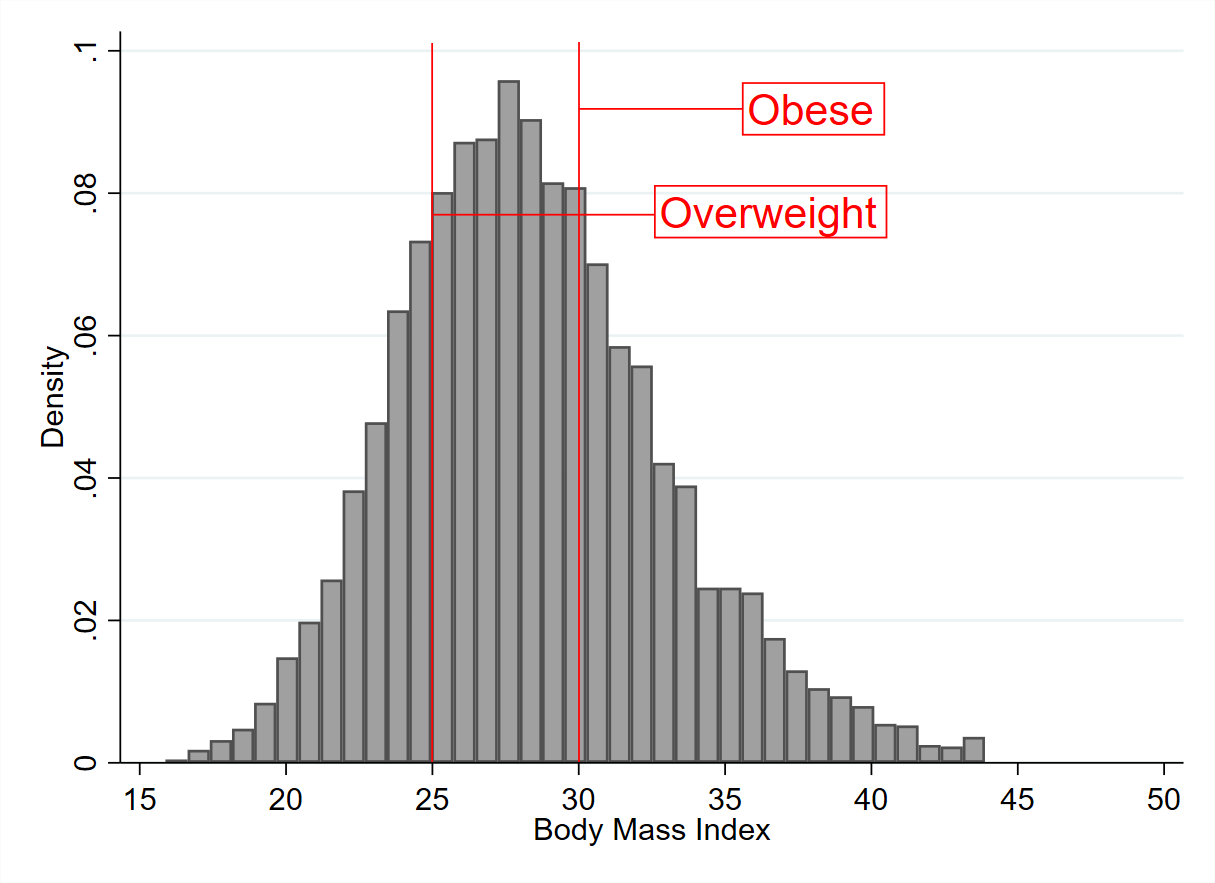
**

**A Further Details on the Creation of Network Buffers**

**A.1. ‘Walkable’ Road Network**

The network buffers, which form the basis of green space analysis in this paper, focus on geographic areas in which the density of local footpaths is, on average, high. GIS techniques were used to extract the relevant set of roads from the full network in our data. The specific steps taken can be outlined as follows: First, roads which were designated as motorways are removed as they cannot facilitate pedestrian access. Then, the length of each remaining road segment, defined as a stretch of road which joins two junctions, was measured. A buffer zone, measuring 25m from each road-segment centreline, was then drawn. Footpath centrelines which fell into each buffer area were associated with the relevant road segment. The length of footpath associated with each road segment was subsequently calculated. Based on these measurements, only roads which satisfied Equation 3 below were included in further analysis.

**A.2. Network Restrictions**

The proximity of each TILDA residential address to the ‘walkable’ road network, as described above, was determined, and green space calculations proceeded as described in the main paper. However, since the generation procedure in Section A.1. created a network which was, necessarily, disjoint in places, we applied a number of further restrictions to our final green space analysis to better capture the areas that were pedestrian-accessible to TILDA respondents. The restrictions are outlined as follows:

- 1. **Distance from the edge of the available data:** We omitted TILDA residential addresses that lay geographically close to the boundaries of the regions for which valid land use data were available. For 1600m buffers, we consider only residential addresses which lay at least 1600m straight line distance from the edge of the data. An equivalent 800m threshold was used when calculating 800m buffers. Since a network buffer is, by construction, smaller than or equal to its radial counterpart in size, this straight line restriction ensured that the shape of the buffer zones in which we analyse green space could not have been influenced by areas where data was missing.
  2. **Urban settlements:** As noted in the main paper, this analysis specifically focused on *urban* green space and, as such, only residential addresses that lay within designated urban settlements as defined by the 2011 Irish national census were considered. It should be further noted that for homes which lay within, but close to the edge of an urban settlement, the green space buffers were, where facilitative roads existed, allowed to extend beyond the settlement boundary to the maximum assumed walking distance (1600m/800m).
  3. **Coastlines:** In coastal areas, it is possible that our GIS process created green space buffers which extended into the sea. Where this occurred, the buffers were clipped at the coastline.
  4. **Network snap distance:** Given that a disjoint road network was used, it was necessary to take steps to ensure that the roads which were assigned as the closest to a residential address accurately represented a feasible starting point from which a given respondent may have begun a walk. To ensure that this assignment was not distorted by the deletion of roads which did not meet the criteria for inclusion in the ‘walkable’ road network (Section A.1.), we considered only residential addresses which were not more than 50m from the nearest road on the final network.
  5. **Total ‘walkable’ space:** For a residence to be included in the green space analysis, the ‘walkable’ road network surrounding a residential address must have been sufficient to allow the full assumed maximum walking distance in at least one direction. In particular, a 1600m buffer must have included at least 1600m of continuous ‘walkable’ road. Equivalently, at least 800m of walkable road was required in a buffer where the maximum distance from the residential address was set to 800m. This restriction ensured a degree of comparability across respondents. TILDA residential addresses that did not meet these criteria are omitted from the green space analysis and were coded in the non-urban category.

**Table S1: Classification of land uses in the vegetation layer of PRIME2**

|  | **Land Function** |
| --- | --- |
| 1 | Athletic track |
| 2 | Bowling green |
| 3 | Burial ground |
| 4 | Cemetery |
| 5 | Cillin [historic burial site] |
| 6 | Field (allotment) |
| 7 | Field (cropland) |
| 8 | Field (pasture) |
| 9 | Field (rough pasture) |
| 10 | Firebreak |
| 11 | Gallops |
| 12 | Golf course |
| 13 | Golf driving range |
| 14 | Golf links |
| 15 | Grassland (pasture) |
| 16 | Grassland (rough pasture) |
| 17 | Graveyard |
| 18 | Green space |
| 19 | Managed woodland |
| 20 | Median |
| 21 | Parkland |
| 22 | Pitch and putt |
| 23 | Pitch (cricket) |
| 24 | Pitch (Gaelic football) |
| 25 | Pitch (hockey) |
| 26 | Pitch (rugby) |
| 27 | Pitch (soccer) |
| 28 | Polo ground |
| 29 | Race track |
| 30 | Rail verge |
| 31 | Road verge |
| 32 | Roundabout |
| 33 | Showground |
| 34 | Sports ground |
| 35 | Sports ground (multiple use) |
| 36 | Tennis court |
| 37 | Traffic island |
| 38 | Unmanaged woodland |
| Note: The dataset also includes a small number of areas which are identified as green but whose land function is unknown. | |

**Table S2: Results using footpath-accessible network buffers**

|  |  | BMI | | | |
| --- | --- | --- | --- | --- | --- |
|  |  | (1) | (2) | (3) | (4) |
|  |  |  |  |  |  |
|  |  | Marginal Effect | Marginal Effect | Marginal Effect | Marginal Effect |
|  |  | (SE) | (SE) | (SE) | (SE) |
| **Share of footpath-accessible Green Space** | |  |  |  |  |
| **1600m Network Buffer** | Non-Settlement | 0.478* | 0.722*** |  |  |
|  |  | (0.272) | (0.238) |  |  |
|  | Quintile 1A (lowest) | 0.800** | 0.722** |  |  |
|  |  | (0.325) | (0.323) |  |  |
|  | Quintile 2A | 0.533* | 0.486 |  |  |
|  |  | (0.298) | (0.299) |  |  |
|  | Quintile 3A | [ref.] | [ref.] |  |  |
|  | Quintile 4A | 0.366 | 0.435 |  |  |
|  |  | (0.320) | (0.320) |  |  |
|  | Quintile 5A (highest) | 0.452 | 0.532* |  |  |
|  |  | (0.319) | (0.318) |  |  |
| **800m Network Buffer** | Non-Settlement |  |  | 0.0764 | 0.269 |
|  |  |  |  | (0.259) | (0.226) |
|  | Quintile 1B (lowest) |  |  | 0.166 | 0.128 |
|  |  |  |  | (0.308) | (0.306) |
|  | Quintile 2B |  |  | -0.199 | -0.226 |
|  |  |  |  | (0.300) | (0.298) |
|  | Quintile 3B |  |  | [ref.] | [ref.] |
|  | Quintile 4B |  |  | -0.0724 | -0.0200 |
|  |  |  |  | (0.311) | (0.312) |
|  | Quintile 5B (highest) |  |  | -0.170 | -0.0739 |
|  |  |  |  | (0.315) | (0.313) |
| **Age Category** | 50-64 | [ref.] | [ref.] | [ref.] | [ref.] |
|  | 65-74 | -0.202 | -0.115 | -0.196 | -0.106 |
|  |  | (0.175) | (0.171) | (0.175) | (0.172) |
|  | ≥ 75 | -0.981*** | -0.843*** | -0.967*** | -0.825*** |
|  |  | (0.236) | (0.214) | (0.236) | (0.214) |
| **Urban Location** | Non-Dublin | [ref.] |  | [ref.] |  |
|  | Dublin | -0.253 |  | -0.158 |  |
|  |  | (0.212) |  | (0.210) |  |
| **Gender** | Male | [ref.] | [ref.] | [ref.] | [ref.] |
|  | Female | -0.902*** | -0.930*** | -0.900*** | -0.928*** |
|  |  | (0.121) | (0.119) | (0.120) | (0.119) |
| **Income Category** | 0 - 9,999 | [ref.] |  | [ref.] |  |
|  | 10,000 - 19,999 | 0.00787 |  | 0.00399 |  |
|  |  | (0.281) |  | (0.281) |  |
|  | 20,000 - 39,999 | -0.0758 |  | -0.0703 |  |
|  |  | (0.256) |  | (0.257) |  |
|  | 40,000 - 69,999 | 0.0605 |  | 0.0615 |  |
|  |  | (0.278) |  | (0.278) |  |
|  | ≥ 70,000 | -0.424 |  | -0.429 |  |
|  |  | (0.316) |  | (0.317) |  |
|  | Not reported | -0.206 |  | -0.185 |  |
|  |  | (0.311) |  | (0.312) |  |
| **Marital Status** | Married | [ref.] |  | [ref.] |  |
|  | Never married | -0.425* |  | -0.422* |  |
|  |  | (0.238) |  | (0.238) |  |
|  | Sep/divorced | -0.374 |  | -0.373 |  |
|  |  | (0.264) |  | (0.265) |  |
|  | Widowed | -0.296 |  | -0.298 |  |
|  |  | (0.202) |  | (0.203) |  |
| **Employment Status** | Employed | [ref.] | [ref.] | [ref.] | [ref.] |
|  | Retired | 0.0252 | 0.140 | 0.0207 | 0.135 |
|  |  | (0.178) | (0.173) | (0.178) | (0.173) |
|  | Other | 0.295* | 0.461*** | 0.290 | 0.457*** |
|  |  | (0.177) | (0.170) | (0.177) | (0.170) |
| **Smoking Status** | Never | [ref.] | [ref.] | [ref.] | [ref.] |
|  | Past | 0.247* | 0.257* | 0.258** | 0.269** |
|  |  | (0.131) | (0.131) | (0.131) | (0.131) |
|  | Current | -1.361*** | -1.331*** | -1.361*** | -1.330*** |
|  |  | (0.185) | (0.183) | (0.185) | (0.183) |
| **Education Level** | Primary/none | 0.485*** | 0.542*** | 0.470*** | 0.530*** |
|  |  | (0.161) | (0.157) | (0.161) | (0.157) |
|  | Secondary | [ref.] | [ref.] | [ref.] | [ref.] |
|  | Third/higher | -0.276* | -0.385*** | -0.294** | -0.402*** |
|  |  | (0.146) | (0.141) | (0.146) | (0.141) |
| **Medical Cover** | Not covered | [ref.] |  | [ref.] |  |
|  | Medical insurance | -0.140 |  | -0.144 |  |
|  |  | (0.215) |  | (0.215) |  |
|  | Medical card | 0.337 |  | 0.343 |  |
|  |  | (0.234) |  | (0.235) |  |
| **Mobility** | No difficulty walking 100m | [ref.] | [ref.] | [ref.] | [ref.] |
|  | Difficulty walking 100m | 1.806*** | 1.849*** | 1.807*** | 1.851*** |
|  |  | (0.299) | (0.299) | (0.299) | (0.299) |
|  | Constant | 28.71*** | 28.31*** | 29.12*** | 28.77*** |
|  |  | (0.413) | (0.265) | (0.403) | (0.258) |
| **N** |  | 5804 | 5807 | 5804 | 5807 |

Standard errors in parentheses. ** p<0.1 ** p<0.05 *** p<0.01*

The results in column (1) refer to the results of the full model with green space footpath-accessibility using a 1600m network buffer. The results in column (2) are those of the parsimonious specification of this model. The results in Column (3) are from full model with green space footpath-accessibility using an 800m network buffer, while the results in Column (4) refer to the more parsimonious specification of this model.

**Table S3: Results using footpath-accessible street-side buffers**

|  |  | BMI | | | |
| --- | --- | --- | --- | --- | --- |
|  |  | (5) | (6) | (7) | (8) |
|  |  | Marginal Effect | Marginal Effect | Marginal Effect | Marginal Effect |
|  |  | (SE) | (SE) | (SE) | (SE) |
| Share of footpath-accessible Green Space | |  |  |  |  |
| 1600m Street-side Buffer | Non-Settlement | 0.285 | 0.466* |  |  |
|  |  | (0.278) | (0.240) |  |  |
|  | Quintile 1C (lowest) | 0.141 | 0.0909 |  |  |
|  |  | (0.329) | (0.322) |  |  |
|  | Quintile 2C | 0.195 | 0.201 |  |  |
|  |  | (0.302) | (0.301) |  |  |
|  | Quintile 3C | [ref.] | [ref.] |  |  |
|  | Quintile 4C | 0.0848 | 0.148 |  |  |
|  |  | (0.315) | (0.312) |  |  |
|  | Quintile 5C (highest) | 0.334 | 0.449 |  |  |
|  |  | (0.338) | (0.331) |  |  |
| 800m Street-side Buffer | Non-Settlement |  |  | 0.256 | 0.427** |
|  |  |  |  | (0.253) | (0.214) |
|  | Quintile 1D (lowest) |  |  | 0.215 | 0.193 |
|  |  |  |  | (0.300) | (0.297) |
|  | Quintile 2D |  |  | -0.0314 | -0.0408 |
|  |  |  |  | (0.294) | (0.294) |
|  | Quintile 3D |  |  | [ref.] | [ref.] |
|  | Quintile 4D |  |  | 0.0550 | 0.128 |
|  |  |  |  | (0.300) | (0.300) |
|  | Quintile 5D (highest) |  |  | 0.190 | 0.309 |
|  |  |  |  | (0.321) | (0.311) |
| Age Category | 50-64 | [ref.] | [ref.] | [ref.] | [ref.] |
|  | 65-74 | -0.182 | -0.0930 | -0.186 | -0.0971 |
|  |  | (0.176) | (0.172) | (0.176) | (0.172) |
|  | ≥ 75 | -0.957*** | -0.814*** | -0.958*** | -0.814*** |
|  |  | (0.236) | (0.214) | (0.236) | (0.214) |
| Urban Location | Non-Dublin | [ref.] |  | [ref.] |  |
|  | Dublin | -0.117 |  | -0.0988 |  |
|  |  | (0.224) |  | (0.214) |  |
| Gender | Male | [ref.] | [ref.] | [ref.] | [ref.] |
|  | Female | -0.901*** | -0.928*** | -0.900*** | -0.927*** |
|  |  | (0.121) | (0.119) | (0.120) | (0.119) |
| Income Category | 0 - 9,999 | [ref.] |  | [ref.] |  |
|  | 10,000 - 19,999 | 0.0371 |  | 0.0175 |  |
|  |  | (0.283) |  | (0.281) |  |
|  | 20,000 - 39,999 | -0.0404 |  | -0.0558 |  |
|  |  | (0.258) |  | (0.256) |  |
|  | 40,000 - 69,999 | 0.0918 |  | 0.0772 |  |
|  |  | (0.279) |  | (0.278) |  |
|  | ≥ 70,000 | -0.399 |  | -0.416 |  |
|  |  | (0.319) |  | (0.317) |  |
|  | Not reported | -0.165 |  | -0.172 |  |
|  |  | (0.313) |  | (0.311) |  |
| Marital Status | Married | [ref.] |  | [ref.] |  |
|  | Never married | -0.406* |  | -0.413* |  |
|  |  | (0.238) |  | (0.238) |  |
|  | Sep/divorced | -0.374 |  | -0.369 |  |
|  |  | (0.265) |  | (0.265) |  |
|  | Widowed | -0.298 |  | -0.296 |  |
|  |  | (0.203) |  | (0.203) |  |
| Employment Status | Employed | [ref.] | [ref.] | [ref.] | [ref.] |
|  | Retired | 0.0295 | 0.143 | 0.0286 | 0.142 |
|  |  | (0.178) | (0.173) | (0.178) | (0.173) |
|  | Other | 0.294* | 0.457*** | 0.293* | 0.457*** |
|  |  | (0.177) | (0.170) | (0.177) | (0.170) |
| Smoking Status | Never | [ref.] | [ref.] | [ref.] | [ref.] |
|  | Past | 0.252* | 0.262** | 0.256* | 0.265** |
|  |  | (0.131) | (0.131) | (0.131) | (0.131) |
|  | Current | -1.362*** | -1.332*** | -1.358*** | -1.328*** |
|  |  | (0.185) | (0.184) | (0.185) | (0.183) |
| Education Level | Primary/none | 0.472*** | 0.530*** | 0.469*** | 0.528*** |
|  |  | (0.161) | (0.157) | (0.161) | (0.157) |
|  | Secondary | [ref.] | [ref.] | [ref.] | [ref.] |
|  | Third/higher | -0.288** | -0.390*** | -0.290** | -0.392*** |
|  |  | (0.146) | (0.141) | (0.146) | (0.141) |
| Medical Cover | Not covered | [ref.] |  | [ref.] |  |
|  | Medical insurance | -0.141 |  | -0.142 |  |
|  |  | (0.215) |  | (0.215) |  |
|  | Medical card | 0.338 |  | 0.339 |  |
|  |  | (0.235) |  | (0.234) |  |
| Mobility | No difficulty walking 100m | [ref.] | [ref.] | [ref.] | [ref.] |
|  | Difficulty walking 100m | 1.809*** | 1.851*** | 1.806*** | 1.848*** |
|  |  | (0.299) | (0.299) | (0.299) | (0.299) |
|  | Constant | 28.87*** | 28.56*** | 28.92*** | 28.61*** |
|  |  | (0.421) | (0.270) | (0.410) | (0.248) |
| N |  | 5804 | 5807 | 5804 | 5807 |

Standard errors in parentheses. ** p<0.1 ** p<0.05 *** p<0.01*

The results in column (5) refer to the results of the full model with green space footpath-accessibility using a 1600m street-side buffer. The results in column (6) are those of the parsimonious specification of this model. The results in Column (7) are from full model with green space footpath-accessibility using an 800m street-side buffer, while the results in Column (8) refer to the more parsimonious specification of this model.

**Table S4: Marginal effects on green space variables for sample excluding non-urban settlements**

|  |  | Marginal Effect |
| --- | --- | --- |
|  |  | (SE) |
| **Share of Footpath-accessible Green Space** | |  |
| **1600m Network Buffer** | Quintile 1A (lowest) | 0.826** |
|  |  | (0.325) |
|  | Quintile 2A | 0.562* |
|  |  | (0.299) |
|  | Quintile 3A | [ref.] |
|  | Quintile 4A | 0.323 |
|  |  | (0.321) |
|  | Quintile 5A (highest) | 0.408 |
|  |  | (0.322) |
| **N** |  | 2243 |

**Table S5: Marginal effects on green space variables in probit model of obesity (BMI>=30)**

|  |  | Marginal Effect |
| --- | --- | --- |
|  |  | (SE) |
| **Share of Footpath-accessible Green Space** | |  |
| **1600m Network Buffer** | Non-Urban Settlement | 0.026 |
|  |  | (0.029) |
|  | Quintile 1A (lowest) | 0.039 |
|  |  | (0.034) |
|  | Quintile 2A | -0.015 |
|  |  | (0.032) |
|  | Quintile 3A | [ref.] |
|  | Quintile 4A | 0.022 |
|  |  | (0.032) |
|  | Quintile 5A (highest) | 0.055 |
|  |  | (0.034) |
| **N** |  | 5804 |

Standard errors in parentheses. * p<0.1 ** p<0.05 *** p<0.01
